# Endocrine Disruptors in Food: Impact on Gut Microbiota and Metabolic Diseases

**DOI:** 10.3390/nu12041158

**Published:** 2020-04-21

**Authors:** Yolanda Gálvez-Ontiveros, Sara Páez, Celia Monteagudo, Ana Rivas

**Affiliations:** 1 Department of Nutrition and Food Science, University of Granada, 18071 Granada, Spain; yolandagalvez@correo.ugr.es (Y.G.-O.); saripaez@correo.ugr.es (S.P.); amrivas@ugr.es (A.R.); 2 Instituto de Investigación Biosanitaria Ibs.Granada, Complejo Hospitalario Universitario de Granada, 18014 Granada, Spain

**Keywords:** endocrine disrupters, food, gut microbiota, metabolic diseases

## Abstract

Endocrine disruptors (EDCs) have been associated with the increased incidence of metabolic disorders. In this work, we conducted a systematic review of the literature in order to identify the current knowledge of the interactions between EDCs in food, the gut microbiota, and metabolic disorders in order to shed light on this complex triad. Exposure to EDCs induces a series of changes including microbial dysbiosis and the induction of xenobiotic pathways and associated genes, enzymes, and metabolites involved in EDC metabolism. The products and by-products released following the microbial metabolism of EDCs can be taken up by the host; therefore, changes in the composition of the microbiota and in the production of microbial metabolites could have a major impact on host metabolism and the development of diseases. The remediation of EDC-induced changes in the gut microbiota might represent an alternative course for the treatment and prevention of metabolic diseases.

## 1. Introduction

It has been widely reported that some exogenous compounds can interfere with the function of the endocrine system in the body. According to the Endocrine Society, an Endocrine Disrupting Chemical (EDC) is “an exogenous [non-natural] chemical, or mixture of chemicals, that interferes with any aspect of hormone action” [1,2]. In this respect, the main source of human exposure to EDCs is food intake. These chemicals might pass into the food chain directly when they are used as pesticides, or they might be released from food packaging containing metals, bisphenol A, or phthalates. In addition, some plant-based compounds (the so-called phytoestrogens) found in dietary supplements also exhibit endocrine disrupting potential [3]. 

Endocrine disruptors have been associated with the increased incidence of metabolic disorders. It has been proposed that EDCs may increase the susceptibility to these disorders by altering the adipose tissue, pancreas, liver, gastrointestinal tract, muscle, and brain homeostatic and hedonic pathways [4]. However, few studies have reported that the effects of EDCs on the gut microbiota can increase the risk of metabolic disorders such as obesity and diabetes [5,6].

Emerging evidence suggests interactions between EDCs and the microbiome, which may affect host health. A key triad between exposure to EDCs, the host genotype and phenotypic responses, and the gut microbiome has been suggested [7]. Exposure to EDCs has been shown to disrupt the microbiome, which may result in dysbiosis and the induction of pathways related to xenobiotics, microbiome-associated genes, enzymes, and the production of metabolites, which may play a crucial role in EDC biotransformation [8]. The products and by-products released following the microbial metabolism of EDCs can be taken up by the host, therefore having an impact on host health and on the development of diseases. In addition, the gut microbiota can modify the EDC profiles through different plausible mechanisms. Microbial enzymes (esterases, thiolases, azoreductases, nitroreductases, β-glucoronidases, methylases, sulfatases, lipases, and β-lyases) can be used to metabolize different types of EDC [9]. Dysbiosis and a reduced diversity of the gut microbiota may cause a reduction in the enzymatic activity, which in turn could result in a decreased metabolization of EDCs to their circulating, active forms, thereby reducing the potential EDC toxicity to the host.

In this work, we conduct a systematic review of the literature in order to identify the current knowledge regarding the interactions between the EDCs in food, thye gut microbiota and metabolic disorders, in order to shed light on this complex triad.

## 2. Methods

The PubMed and Web of Sciences databases were searched to identify the relevant studies. The following keywords were used: “Gut microbiota”, “Diet”, “Obesity”, “Diabetes”, “Bisphenol A”, “Bisphenol A analogs”, “Microbiota”, “Pesticides”, “Parabens”, “Polychlorinated biphenyls”, “Phytoestrogens”, “Metals”, “Cadmium”, “Arsenic”, “Lead”, “Phthalates”, “Triclosan”, and “Triclocarban”. Data published between 2006 and 2020 were considered.

**Figure 1:**
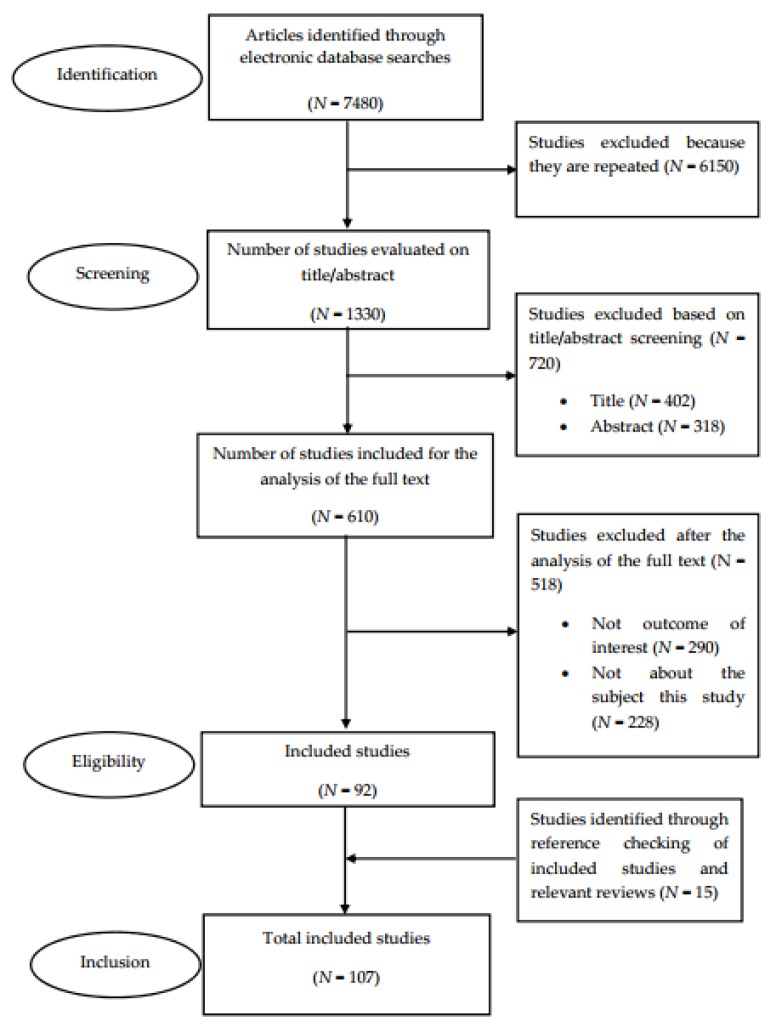
A flow diagram of the literature search.

The literature review was conducted in compliance with the recommendations provided in the Preferred Reporting Items for Systematic Reviews and Meta-Analyses (PRISMA). Figure 1 shows the PRISMA flow diagram that maps out the number of studies identified, those included and excluded, and the reasons for exclusion ((a) papers not written in the English language, (b) no outcome of interest, and (c) not related to the subject of the study). A total of 107 studies were included for analysis [10].

## 3. Results and Discussion

### 3.1. The Gut Microbiota in Health and Metabolic Diseases

A remarkable amount of evidence has emerged in recent years that strongly suggests that an essential role is played by the human microbiota in health and disease development via several mechanisms [11]. Variations in gut microbiota composition are considered to be physiological from the perspective of healthy gut microbiota, and these changes are related to age; sex; and external factors such as dietary habits, exercise, and antibiotic use. Indeed, dysbiosis, defined as the alteration of gut microbiota communities, is often related to health disorders. 

*Firmicutes* and *Bacteroidetes* are the two primary phyla in gut microbiota, accounting for 90% of the total composition [12]. Other phyla include *Actinobacteria*, *Proteobacteria*, *Fusobacteria*, and *Verrucomicrobia* [13]. The *Firmicutes* phylum is composed of more than 200 different genera such as *Enterococcus*, *Lactobacillus*, *Ruminicoccus*, *Bacillus*, and *Clostridium*. The phylum *Bacteroidetes* is dominated by the genera *Prevotella* and *Bacteroides.* The *Actinobacteria* phylum is comparably less abundant and mostly represented by the *Bifidobacterium* genus [13].

In recent years, research has demonstrated that gut microbiota could play an important role in the pathophysiology of metabolic disorders, specifically in obesity and diabetes [14]. Animal studies have shown that obesity is related to changes in the gut microbiota composition, including a reduction in species variety and alterations in the genes involved in metabolism. By contrast, data related to the human microbiota are more variable [13]. When fed with a similar diet, the comparison of the gut microbiota of genetically obese (ob/ob) mice with that of lean mice showed a greater abundance of *Firmicutes* and lower relative abundance of *Bacteriodetes* (50%) in obese mice [15]. These changes in the microbiota have also been found in different human studies [16,17]. Moreover, low relative amounts of *Bifidobacterium vulgatus* and high abundances of *Lactobacillus* spp. are detected in the microbiota of obese children [18]. Other studies have reported on the relationship between the *Proteobacteria* phylum and obesity, by the identification of pro-inflammatory molecules such as lipopolysaccharides and increased fat storage in the host [19]. In addition, higher abundances of *Rikenellaceae* and *Ruminococcaceae* have been revealed in leptin-resistant obese (leptin-promoting satiety) and diabetic (db/db) mice compared with in the lean members of the same litter [20]. Furthermore, other authors have demonstrated a relationship between *Desulfovibrionaceae* growth induction, obesity, and type 2 diabetes (T2D) [21,22]. 

There is also cumulative evidence from human and animal studies of an association between the development of diabetes and the existence of changes in the gut microbiota composition. Larsen et al. (2010) [23] showed that the *Firmicutes* phylum and *Clostridia* class decreased significantly in humans with T2D compared to in the healthy control group. Likewise, the β-*Proteobacteria* class increased in diabetics compared to in the control group and was positively associated with plasma glucose levels. Murri et al. (2013) [24] reported increased *Veillonella, Bacteroidetes*, and *Clostridium* spp.—along with a decrease in *Blautia, Lactobacillus*, *Prevotella*, and *Bifidobacterium*—in children with diabetes type 1 diabetes (T1D) compared to in the healthy control group.

### 3.2. Role of EDCs in the Microbiota

Table 1, Table 2 and Table 3 provide information concerning the effect of endocrine disruptors on gut microbiota in vitro, in animals, and in human studies.

#### 3.2.1. BPA and Analogs

Bisphenol A (BPA) is an environmental chemical widely used in industry for the manufacture of polycarbonate plastics and epoxy resins, with well-known endocrine disrupting activity [25,26]. The public concern about the safety of BPA has resulted in the imposition of a ban on its use in some products and the emerging market entry of BPA analogs such as bisphenol S (BPS), bisphenol F (BPF), bisphenol AF (BPAF), and bisphenol B (BPB). However, their structural similarity to BPA has also raised concerns about their endocrine disrupting potential. Several studies have reported on the association of BPA and BPA analogs with an increased risk of developing metabolic diseases such as obesity [25,27]. However, this association is not well demonstrated, and it is challenging to find evidence for direct causality between BPA and analog exposure and metabolic diseases using epidemiological studies. 

Wang et al. (2018) [28] studied the changes in metabolism and accessibility of BPA in different parts of the gastrointestinal tract using an in vitro Simulator of the Human Intestinal Microbial Ecosystem (SHIME) model. Three different BPA concentrations were investigated, which provided information regarding an extensive range of BPA daily intake values, from the human relevant exposure dose (25 μg/L) and the EPA (Environmental Protection Agency) reference dose (250 μg/L), to the 1% lowest observed adverse effect level (2500 μg/L) [28]. The toxicity of BPA, in terms of the effects on the hepatic gene expression profiles, was compared with that of the SHIME effluents, using the human hepatocellular carcinoma (HEPG2) cell line. The findings showed that BPA exposure modified the microbial composition of the colon, increasing the amount of microbes in the ascending, transverse, and descending colon. The upregulation of BPA-degrading bacteria, such as *Microbacterium* and *Alcaligenes*, was also reported.

Exposure to BPA and BPA analogs in animal models such as rodents, zebrafish, rabbits, and dogs can affect the gut microbiota and have an impact on the development of metabolic diseases. Some studies have reported that there is a sexual dysmorphic effect [6,29,30,31,32,33].

Xu et al. (2019) [33] determined the impact of BPA exposure on the development of T1D and the involvement of the host immune system and gut microbiota in a model of non-obese diabetic (NOD) mice. Adult male and female NOD mice were orally exposed to BPA at environmentally relevant doses (30 or 300 µg/kg). These doses were selected because they had previously been shown to modify the immune system and to be relevant for human exposure (30 BPA/kg body weight (bw) is within the range of human exposure levels, and 300 BPA/kg bw is also appropriate for human exposure levels based on BPA concentrations in human blood) [34]. In addition, the current EPA reference dose is 50 μg/kg/day. However, exposure to low-dose BPA also seems to have harmful effects, and as a consequence, after careful examination, the European Food Safety Authority (ESFA) has lowered the total dietary intake to 4 μg/kg bw/day.

Exposure to BPA resulted in a fast onset of T1D in female mice and slow onset in male mice. Subacute BPA exposure in female mice resulted in increased *Bacteroidetes* and *Cyanobacteria* and decreased *Firmicutes, Tenericutes*, and *Proteobacteria.* Chronic exposure to BPA in females also resulted in a proinflammatory gut microbiota, with a decrease in *Bacteroidales* and *Lactobacillus*. These results are consistent with human epidemiological studies, which show that the gut microbiota in individuals with T1D is dominated by *Bacteroidetes* at the phylum level and in turn with a reduction in *Firmicutes* in relation to control [87]. In male mice, BPA exposure results in a slow onset of T1D. Subacute exposure caused a decrease in *Bacilli* at the class level, which is in accordance with the findings reported in human epidemiological studies on the association between the the development of T1D and gut microbiota composition [88]. Additionally, chronic exposure to BPA in males resulted in decreased *Lachnospiraceae,* which has been shown to promote T1D in NOD mice models [89].

Using their NOD mouse model, Xu et al. (2019) [6] showed that BPA’s effects on the development of T1D were related to host age and gender, following various windows of exposure. Exposed juvenile NOD females (starting postnatal day (PND) 28 to PND56) and NOD offspring (starting gestation day 5 to PND28) were exposed perinatally to BPA by dosing the dams to 0 or 30 µg/kg BW. Adult NOD females were exposed to 0 or 300 µg/kg bw. Interestingly, BPA increased the risk of developing T1D in adult and juvenile females, which was related to changes in the gut microbiota, but the female offspring showed a reduced risk of developing T1D. In contrast, BPA had insignificant effects on the development of T1D in the male offspring. The changes in the gut microbiota of juvenile females associated with BPA exposure included, at the genus level, an increase of *Turicibacter, Oscillospira*, *Ruminococcus, Jeotgalicoccus*, and *Lachnospiraceae*, which increase the risk of T1D and inflammation, as shown in several animal models [89,90,91].

Malaisé et al. (2017) [5], in their longitudinal study, found that the perinatal exposure of C3H/HeN mice to BPA at 50 µg/kg bw/day (100 times lower the no observed adverse effect level (NOAEL), 5 mg/kg bw/day) induced dysbiosis and systemic immune imbalances at PND45. These effects were associated with a rise in glucose intolerance and a decrease in IgA and *Bifidobacteria* in the feces. Some strains of the *Bifidobacterium* genus have been shown to have anti-inflammatory properties [92].

Javurek et al. (2016) [31] explored the changes in gut microbiota related to BPA exposure in parents and their offspring in California mice (*Peromyscus californicus*). Female and male monogamous and biparental mice were exposed to BPA (50 mg/kg feed weight) from periconception to weaning. The dose selected has been shown to induce metabolic alterations and it is below the diet-administered maximum non toxic dose for rodents (200 mg/kg BW/day). This dose is within the presumptive NOAEL and results in concentrations similar to those reported in human serum. They demonstrated for the first time that parental exposure to concentrations of BPA environmentally relevant causes changes in the microbiota structure in non-exposed offspring. These changes were generational- and sex-dependent. In this respect, they reported that BPA (and ethinyl estradiol) exposure induced an increase in *Akkermansia, Mollicutes, Prevotellaceae, Bacteroides*, *Erysipelotrichaceae, Methanobrevibacter, Sutterella* in parents and offspring. These species have been associated to inflammatory bowel disease, obesity and metabolic disorders [93,94], autism spectrum disorders [95], colon cancer [96], and other conditions. However, *Bifidobacterium* was also found in higher amounts in fecal samples of female offspring. Some *Bifidobacterium* strains have been shown to exert health-promoting effects and is included in a number of probiotic foods and supplements [97]. DeLuca et al. (2018) [46] used a dextran sulphate sodium-induced colitis model in female C57BL/6 mice and found that BPA exposure at 50 μg/kg/day negatively affects gut physiology by reducing microbiota metabolites derived from aromatic amino acids, which might be associated with autoimmune diseases, specifically with inflammatory bowel disease. This dose was selected because it is the EPA reference dose for BPA.

Lai et al. (2016) [48] used 16S rRNA gene sequencing of cecal microbiota of CD-1 male mice to analyse the effects of dietary BPA intake on microbiota composition and physiology. Mice on high-fat high-sucrose diet were the positive controls. The findings showed that dietary BPA exposure was related to a decrease in the diversity of microbiota species. The structural changes of the gut microbiota exposed to dietary BPA were similar to those found in mice on high-fat high-sucrose diets. Additionally, the comparison between BPA and high-fat diet revealed an increase in *Proteobacteria* in both groups. The increased abundance of *Proteobacteria* has been related to different conditions such as metabolic disorders and inflammatory bowel disease [98]. Lastly, exposure to dietary BPA produced a decrease in the phylum *Firmicutes*, with most of the 16SRNA sequencing corresponding to the class *Clostridia*. Interestingly, Larsen et al. (2010) [23] demonstrated a significant reduction in *Firmicutes* and *Clostridia* in the feces of human male adults with T2D compared to the healthy group. 

Dietary exposure to BPA also modified the gut microbiota in zebrafish [30,32]. In a study conducted by Liu et al. (2016) [32], the authors concluded that exposure to BPA resulted in an increase of the phylum CKC4 in both sexes probably connected to changes in the host lipid metabolism (increased triglycerides in the muscle). However, one of the limitations of this study was that the functional study of CKC4, a phylum included in the SILVA database, was very incomplete.

Chen et al. (2018) [30] exposed adult zebrafish at BPA doses of 0, 2 and 20 μg/L, titanium dioxide nanoparticles (nano-TiO_2_) and their binary mixtures for three months. Exposure to both compounds resulted in changes in the gut microbiota. An antagonistic interaction was observed at low BPA concentrations, but a synergistic interaction was observed at high BPA concentrations. Zebrafish growth and gut health (oxidative stress, barrier function, inflammation) were associated with sex and concentration of chemicals. Additionally, zebrafish weight was found to be positively associated with the presence of Bacteroides, closely related to the *Anaerococcus*, *Finegoldia*, and *Peptoniphilus* genera.

The work by Reddivari et al. (2017) [49] showed that perinatal exposure to BPA in Dutch-Belted rabbits (200 μg/kg bw/day) induced an increase in the *Methanobrevibacter* spp community leading to inflammation of the colon and liver, and increased gut permeability in the offspring demonstrated by increased levels of serum lipopolysaccharide. This study used rabbits as an animal model because this species has an extensive infantile period of development similar to that of humans and utilized BPA at a relatively low dose level of 200 g/kg bw/day (approximately 1/25 of the NOAEL dose). Significant positive correlations were observed between increased *Methanobrevibacter* spp. in the colon and systemic concentrations of lipopolysaccharide (*r*^2^ = 0.67; *p* = 0.023). This species can metabolize dietary substrates that lead to increased host energy intake and weight gain [93]. Lastly, perinatal exposure to BPA led to a reduction of the diversity of the microbial communities and their metabolites (short chain fatty acids) and to an increase in intestinal permeability.

The work by Koestel et al. (2017) [47] showed that the circulating BPA levels in dogs fed with canned dog food for two weeks (2.2 ± 0.15 ng/mL) were similar to the levels found in humans [99], with higher BPA concentrations related to modifications in the composition of the microbiome. These changes may lead to modifications in metabolic pathways, including the capacity to metabolize bisphenols. In this study, higher serum BPA levels were associated with decreasing relative abundances of *Bacteroides* spp., which may result in a reduction of BPA bacterial degradation. 

We found only one study of the effects of BPA analogs on the microbiota. Catron et al. (2019) [29] exposed zebrafish during their developmental period to BPA, BPAF, BPB, BPF, and BPS at different concentrations. These concentrations were selected based on zebrafish toxicity data available through the ICSS ToxCast dashboard and previous zebrafish studies. 16S rRNA gene sequencing showed that structural microbiota disruption was highly dependent on concentration and that exposure to BPS, BPA, or BPF caused the enrichment of the microbial functions, but this did not occur with exposure to BPB or BPAF. Lastly, microbial disruption was inversely associated with host developmental toxicity and estrogenicity. The main finding of this work was that BPS, BPA, and BPF produced disruptions in microbial composition, occurring throughout the critical window of early development, at concentrations that did not cause evident developmental toxicity. 

#### 3.2.2. Pesticides

The adverse effects of agricultural pesticides derived from their endocrine disruptive activity that may affect the thyroid and the reproductive, nervous, and adipose systems were investigated [100,101,102].

In vitro studies have demonstrated that different pesticides can cause gut dysbiosis [38,39,40,41], using the chicken microphrobiome [39] and the Rumen Simulation Technique (RUSITEC) system [40,41]. Joly et al. (2013) [38] studied the impact of in vitro exposure to low doses of organophosphorus chloropyrifos in a SHIME system, and the effects in vivo in pregnant Wistar rats. Their results showed that exposure to chlorpyrifos induces the proliferation of *Bacteroides* spp. and *Enterococcus* spp. and reduces the proliferation of *Bifidobacterium* spp. and *Lactobacillus* spp.

Three studies in rodents (in ICR, C57Bl/6, and CD-1 mice and Wistar rats) showed that exposure to pesticides (carbendazim, chlorpyrifos, and organophosphorus pesticides) induces dysbiosis in the microbiota and inflammation, leading to an alteration of lipid metabolism and triggering obesity in exposed rodents [69,70,73].

Wu et al. (2018) [74] evaluated the effects of exposure to propamocarb (3, 30, and 300 mg/L for 28 days) in five week-old male Institute of Cancer Research mice. These concentrations were established based on the highest residue from the EU-Maximum Residues levels and the long-term toxicity NOAEL (20 mg/kg bw/day) [103]. The authors found that exposure induced the disruption of the transcription of hepatic genes involved in the regulation of lipid metabolism. The cecal and fecal microbiota changed at the phylum or genus levels. 

Liu et al. (2017) [71] exposed adult male C57BL/6 mice, over eight weeks, to low dose p,p’-dichlorodiphenyldichloroethylene (1 mg/kg body weight/day) and β-hexachlorocyclohexane (10 mg/kg body weight/day), which are similar to the levels found in chronic exposure in humans. The authors found that exposure induced changes in the composition of the gut microbiota, particularly leading to higher abundances of *Lactobacillus* that are capable of deconjugating bile acids by bile salt hydrolases. These transformation reactions affect the hydrophobicity and composition of bile acids, and down-regulate the expression of genes involved in the reabsorption of bile acids in the distal ileum, but up-regulate the expression of genes involved in the hepatic synthesis of bile acids. 

Tu et al. (2019) [72] evaluated the toxic effects of exposure to 2,4-dichlorophenoxyacetic acid, at an occupationally important dose, on the intestinal microbiota of specific-pathogen-free C57BL/6 male mice. Metagenomic sequencing showed a distinct gut microbial community with disturbances in the pathways of amino acid and carbohydrate metabolism and urea degradation. These findings are of particular interest, as evidence has showed that modifications of microbiome-related pathways and metabolites would produce an alteration of gut-host homeostasis, which may increase the risk of diseases [104].

The sexual dysmorphic effects of pesticide exposure have been described in animal models. Gao et al. (2017) [68] showed that exposure to a low dose of organophosphorus pesticide diazinon (4 mg/L in drinking water) modifies the gut microbiota composition and functionality in C57BL/6 mice, with more impact in male mice. At the phylum level, *Bacteroidetes* increased by 1.8-fold, while *Firmicutes* decreased by 1.8-fold, in diazinon exposed males compared to controls. As shown previously, a high *Firmicutes*/*Bacteroidetes* ratio is related to obesity, which is consistent with the observed decrease of body weight in male mice. By contrast, no effects of diazinon exposure on body weight and the *Firmicutes*/*Bacteroidetes* ratio were observed in female mice. 

Finally, an epidemiological study in humans carried out by Stanaway et al. (2017) [84] found that exposure to agricultural pesticides can cause dysbiosis of the human oral microbiota. A cohort of 65 agricultural workers and 52 non-agricultural workers was studied. The results showed that workers exposed to azinfos-methyl had a decrease in common genera found in the human oral microbiome (*Streptococcus, Micrococcineae, Gemella, Haemophilus, Halomonas, Actinomycineae*, and *Granulicatella*). Although more studies are needed to confirm the results of Stanaway et al. (2017) [84], the data obtained indicate that the oral microbiome could be used as a simple biomarker for assessing pesticide exposure in epidemiological studies.

#### 3.2.3. Polychlorinated Biphenyls

Polychlorinated biphenyls (PCBs) are well-known EDCs that were massively used until the mid-1970s as insulators for electrical equipment such as transformers, switches, capacitors, and thermostats. Despite the current ban on manufacturing, PCBs continue to be a common environmental contaminant because of their accidental and intentional release in large quantities due to their long-term stability [105,106]. The main route of exposure to PCBs is the consumption of contaminated foods such as fish and shellfish. PCBs have been related to hormone-dependent cancers, an impaired reproductive system, and cognitive and metabolic disorders (such as impaired glucose metabolism and adipocyte inflammation) [107,108]. 

The available evidence has shown the detrimental effects of PCB exposure on the microbiota that can trigger host metabolic disorders [36,50,51,52,53,54,55,109]. Exposure to PCBs promotes an altered microbiome, with reduced *Proteobacteria* [53], decreases in microbial diversity, and an increased *Bacteroidetes*-to-*Firmicutes* ratio, usually linked to intestinal and systemic inflammation [55]. In addition, changes in bile acid homeostasis that result in dysbiosis have been described in adult mice exposed to a PCB mixture [51]. Other studies have also reported the impact of exposure to PCB on amphibian [54] and zebrafish microbiomes [50]. Recently, Rude et al. (2019) [56] reported that developmental exposure to PCBs induces dysbiosis and epithelial permeability defects in the ileum and colon that result in changes to the microbial β-diversity of juvenile mice.

Petriello et al. (2018) [55] found that exposure to PCB126 (1 mmol/kg) induces disruption of the gut microbiota and host metabolism in seven week-old male Ldlr −/− mice, which are a model of cardiometabolic disease. This PCB126 dose was selected because it results in plasma concentrations similar to those found in human exposure [110]. The 16S rRNA sequencing revealed changes at the phylum and genus levels, and consistent increases in intestinal and systemic inflammation. The *Firmicutes*/*Bacteroidetes* ratio was increased after PCB126 exposure. This increase has been consistently linked to obesity, insulin resistance, inflammation, and other alterations of host metabolism [111]. Additionally, a strong PCB-dependent association was found between *Bifidobacterium* and circulating glucagon-like peptide-1.

Chi et al. 2019 [52] exposed adult female C57BL/6 mice to environmentally relevant low-dose PCB126 (50 μg/kg bw) once per week over six weeks and found that chronic low-dose exposure promoted dysbiosis, with changes to the microbiota’s structure and composition. In addition, PCB126 promoted dyslipidemia, hepatic damage, and a fatty liver. Lastly, metabolic indicators of these conditions seem to positively correlate with specific bacterial taxa. 

The study by Chen et al. (2018) [50] showed that the exposure of zebrafish to model pollutants with different mechanisms of action and different affinities to estrogen and aryl hydrocarbon receptors (atrazine, estradiol, PCB126, and PCB153) at 1.0 μg/L for seven days resulted in changes to the microbiota and in the deterioration of intestinal and hepatic functions. The authors reported that aryl hydrocarbon and estrogen receptor signaling regulate gut microbiota physiology. Data showed that impaired *Aeromonas* reproduction was significantly related to oxidative damage, especially in the PCB126 groups. Previous research has linked *Aeromonas* spp. to intestinal inflammation and soft tissue infection [112]. 

#### 3.2.4. Parabens

Parabens are widely used as preservatives and bactericides in pharmaceuticals, personal care products, and some food products [113,114,115]. Several studies have reported on the adverse health effects of parabens, including their endocrine disrupting and obesogenic activities [116,117,118,119]. However, there are few data available regarding the effects of parabens on the microbiota.

In this respect, Hu et al. (2016) [66] analyzed the effect of low-dose exposure to methylparaben, triclosan, and diethylphthalate and their mixtures, as well as the window of susceptibility, in Sprague-Dawley rats. The doses investigated result in urinary biomarker levels similar to those observed in humans [120]. The *Bacteroidetes* phylum was increased, while the growth of *Firmicutes* was decreased in all exposed rats compared to controls. However, *Betaproteobacteria* was increased only in the methylparaben and mixture groups, suggesting that exposure to paraben mixtures produces a distinct microbiome shift different from that resulting from individual chemicals or from simple additive effects. Surprisingly, these differences decreased in adulthood. In addition, a reduction in body weight in rats exposed during adolescence was observed [66]. This is consistent with other studies reporting a decrease in the *Bacteroidetes* phylum and an increase in *Firmicutes*, linked to weight gain [121,122]. These studies highlight i) the importance of studying the critical window of exposure such as adolescence; ii) the effect of low dose exposure, similar to a human exposure scenario; and iii) the need to evaluate the combined effect of multiple exposures. Obadia et al. (2018) [65] observed that exposure to methylparaben (0.1% and 0.3%) in fly medium reduces microbiota growth and modifies the composition and amount of the bacteria and yeasts in the intestine of the *Drosophila* fly.

The effects of parabens on the microbiota need further research, as their interactions are poorly understood.

#### 3.2.5. Phytoestrogens

Phytoestrogens are compounds naturally occurring in plants that have estrogenic/antiestrogenic effects. Phytoestrogens can modulate and be metabolized by the gut microbiota [123]. Phytoestrogen activity is strongly dependent on the microbiome. Their metabolites have stronger estrogenic activity than the natural compounds themselves, and because of the variability in microbiomes, there are large differences in the effects of phytoestrogens among individuals [124,125].

Daidzen, a phytoestrogen present in soy-based foods, can be metabolized to O-desmethylangolensin (ODMA) and equol by gut microbial communities in 80–95% and 25–60% of the population, respectively. In relation to this, Frankenfeld et al. (2011, 2014) [126,127] evaluated the presence of ODMA- and equol-metabolizing phenotypes in obese, overweight, and normal-weight individuals and found that the ODMA-metabolizing phenotype, but not the equol-phenotype, was linked to obesity in adulthood.

Another study investigated the effects of *S*-equol on pancreatic β-cell growth and insulin secretion in male mice. The results showed that *S*-equol boosts β-cell function and prevents hypoglycemia in mice, suggesting that *S*-equol may act as a potential preventive agent against type 2 diabetes mellitus [57]. Zhou et al. (2018) [64] investigated whether genistein intake by C57BL/6 female mice can reduce the negative impact of a maternal fat-high diet on glucose and lipid metabolism in their offspring. Female mice were placed on a high-fat diet alone, a high-fat diet supplemented with genistein at low (0.25 g/kg diet) and high doses *n* (0.6 g/kg diet), or a genistein-free control diet, for three weeks prior to pregnancy and throughout gestation and lactation. After weaning, female offspring from the high-fat group had lower weight at birth, as well as glucose intolerance and higher insulin, triacylglycerol, and total cholesterol levels in the serum compared with the control group. Offspring from the low-dose genistein group showed an increased weight at birth, improved glucose tolerance, and decreased fasting insulin. Offspring from the high-dose genistein group showed decreased serum triacylglycerols and total cholesterol compared with the offspring from the low-dose genistein mothers. The high abundances of *Bacteroides* and *Akkermansia* in the offspring from the genistein-fed female parents might be key to the improvement of glucose metabolism. A decrease of *Bacteroides* has been shown in diabetes patients in comparison to in healthy controls [128]. In addition, it has been reported that *Akkermansia* could preserve the mucus layer thickness and is correlated with an improved metabolic profile [129]. Similarly, the increased *Rikenella* in the offspring from the high genistein group might be linked to the decreased triacylglycerols and total cholesterol in the serum. In addition, it has been reported that the abundance of *Rikinella* contributes to a lean body type phenotype [130].

Lopez et al. (2018) [59] also found, in nine-week-old male C57/BL6 mice, lower serum triglycerides, improved glucose metabolism, and lower weights in the high-fat diet and genistein group (3 mg/kg/day) compared to in mice fed the high-fat diet alone. In addition, the presence of genistein in the high-fat diet resulted in changes to the gut microbiota (increases in the *Prevotella* and *Akkermansia* genera), linked to lower circulating levels of lipopolysaccharides and the reduced expression of pro-inflammatory cytokines in the liver, compared to in mice in the high-fat diet alone group. It has been showed that the reduction of lipopolysaccharides can decrease neuroinflammation [131].

Recently, Huang et al. (2018) [58] investigated, in non-obese diabetic mice, perinatally exposed to physiological doses of genistein (20 mg/kg body weight), whether there is a sex-dependent effect on type 1 diabetes (T1D). In female offspring, perinatal exposure to genistein resulted in a higher incidence of early-onset T1D. In addition, increased *Enterobacterials* were found in the fecal microbiota from the PND90 female offspring, which is indicative of a pro-inflammatory response. These changes were not found in the PND30 females. However, perinatal genistein exposure in PND90 males induced changes in the gut microbiota linked to an anti-inflammatory response. The authors conclude that a strong sex-specific effect was found in the perinatal genistein exposure window and that the mechanism of T1D in non-obese diabetic females is induced by immune system modulation of the gut microbiota. These results must be taken into account, since soy milk formula consumption during infancy was related to type 1 diabetes [132].

In California mice (Peromyscus californicus), Marshall et al. (2019) [60] also determined whether perinatal exposure to genistein (250 mg/kg feed weight) promoted dysbiosis and altered gut metabolites. Female mothers were fed a diet with genistein or a genistein-free control diet. Their results showed that exposure to genistein resulted in sex-related disturbances of the gut microbiota and metabolites in the offspring. Positive associations between the gut microbiome, metabolome, and disruption of social and vocalization behaviors (audible calls above 20 kHz) were also found in the offspring of exposed dams. When male offspring from genistein-supplemented dams were compared with genistein-free offspring, calls above 20 kHz correlated with daidzein, α-tocopherol, *Flexispira* spp., and *Odoribacter* spp. The effects secondary to genistein exposure may result from disturbances to neurobehavioral programming or from changes in the microbiota linked to changes in gut metabolites. These results suggest that the gut microbiome and its metabolites can induce a disruption in the offspring’s neurobehavioral programming, known as the “microbiome gut–brain axis”.

The effect of soy intake on microbiota composition and diversity has been studied in other animal models, such as porcine models [61,63] and the Southern white rhinoceros [62]. Yeruva et al. (2016) [63] determined the influence of diet on the development of the immune system in neonates, using a porcine model. Two-day old piglets were fed soy or milk formula until day 21 and compared to a sow-fed group, and the results showed that the formula diets induced changes in the small intestine microbiome, particularly in the duodenum. Significant increases in *Lactobacillaceae* spp. and *Clostria* spp., as well as a decrease in *Enterobacteriaceae* spp., were found in soy-fed piglets.

There is, however, little information regarding the impact of phytoestrogens on the human microbiota. Wu et al. (2016) [82] compared the plasma metabolites in omnivores versus vegans that consume soyfoods and found significant differences in the metabolomes between the two groups, but the gut microbiota was very similar in both groups [82].

#### 3.2.6. Metals

Metals have been considered EDCs because of their ability to bind to hormone receptors [133]. Metals are ubiquitous environmental pollutants, with the primary sources of human exposure being the inhalation of dust or direct ingestion of contaminated food and water. Metal exposure has been related to obesity, diabetes, and metabolic syndrome [134,135,136,137].

Metals can be metabolized by the colonic microbiota in humans. Van de Wiele et al. (2010) [35] reported the ability of this microbiota to methylate Arsenic (As), which suggests that the role played by microbiota metabolism should be considered when assessing the toxic effects on human health of ingested As.

Lu et al. (2014) [45] reported that the composition of the gut microbiome in C57BL/6 mice markedly alters after exposure to 10 ppm arsenic in the drinking water over 4 weeks, resulting in a decrease in four *Firmicutes* families. These results are in agreement with the As antiobesogenic properties described in several articles [138]. They also reported a significant association between this microbiota disruption and changes in microbiota metabolites. This suggests that exposure to As not only induces disturbances in the abundance and composition of bacterial communities but also affects their metabolomic profile, which subsequently results in the disturbance of host metabolite homeostasis. These changes in metabolite homeostasis are important risk factors involved in tissue dysfunctions, which may cause diseases such as obesity and diabetes [139].

Wu et al. (2016) [43] reported that perinatal lead (Pb) exposure (32 ppm) in the drinking water, in wild-type non-agouti (a/a) mice of the Avy strain isogenic mouse model of perinatal environmental exposure, induced changes in the adult offspring gut microbiota. These changes were sex-independent, but a strong association was found between male offspring and increased body weight. Interestingly, the quantities of the two predominant phyla (*Bacteroidetes* and *Firmicutes*) shifted inversely with Pb exposure. In addition, reduced aerobic bacteria and increased anaerobic bacteria were observed in the exposed offspring. Lastly, *Pseudomonas*, *Enterobacter*, and *Desulfovibrio* were found in higher abundances in exposed adult mice than in controls (*p* < 0.05) [43].

Ba et al. (2017) [42] demonstrated the sex-specific effects of low-dose exposure to cadmium and found that early exposure to 100 nM induced fat accumulation in adult male C57BL/6J mice. In this work, 100 nM cadmium was present in the drinking water, which is equivalent to ~ 2.5 μg/kg bw per week and corresponds to the tolerable weekly intake and the mean intake by humans [140]. They also found an increased metabolism of fatty acids and lipids as well as a decrease in the composition and diversity of the gut microbiota. At eight weeks, the gut microbiota was found to be particularly vulnerable to low-dose cadmium exposure, and exposure during this period may induce adiposity in adult mice, even if the microbiota is later restored. The role played by the gut microbiota in adiposity related to cadmium exposure was also demonstrated by microbiota transplantation and removal experiments.

Xia et al. (2018) [44] observed that short-term exposure to 10 and 30 μg/L Pb increased the volume of intestinal mucus in the adult male zebrafish. They also found decreased α-*Proteobacteria* and increased *Firmicutes* after exposure to 30 μg/L Pb for seven days. In addition, 16S rRNA sequencing demonstrated an altered gut microbiota, in terms of composition and diversity, after exposure to 30 μg/L Pb. At this dose, 52 gut microbes and 41 metabolites underwent significant changes, particularly those related to the pathways of glucose, lipid, amino acid, and nucleotide metabolism. Lastly, they also found a marked reduction in the transcription of some genes related to glycolysis and lipid metabolism after seven-day exposure to 30 μg/L Pb.

A recent study showed that the urinary concentrations of Pb in adult humans were related to changes in gut microbial composition, even at low Pb levels [81], and so an association between increased urinary Pb concentrations and increased microbiota α-diversity and richness was found. Changes in β-diversity were significantly associated with changes in urinary Pb concentrations, and *Proteobacteria*, including members of the *Burkholderiales* (a wide variety of bacterial species that perform a plethora of metabolic functions), were also associated with increased urinary Pb [81].

#### 3.2.7. Triclosan and Triclocarban

Triclosan (TCS) and triclocarban (TCC) (TCs) are chlorinated, broad-spectrum antimicrobial endocrine disrupting chemicals found in thousands of consumer and industrial products [141], as well as in contaminated food [142,143]. These EDCs have been related to metabolic disorders such as obesity and diabetes [144,145].

It has been shown that TCS exposure induces changes in the gut microbiota of rats [66,77], mice [75], and fish [76,79]. Narrowe et al. (2015) [79] showed that even low but environmentally relevant levels of triclosan exposure (100–1000 ng/mL) can result in the disturbance of the juvenile fish gut microbiome. Seven-day exposure to triclosan in larval fathead minnows (*P. promelas*) resulted in significant changes, as measured by α- and β-diversity, in the gut microbiome immediately after triclosan exposure; however, the microbiome rapidly recovered following two weeks of depuration. This demonstrates the sensitivity and resilience of the gut flora to the toxic effects of environmental contaminants.

Kennedy et al. (2016) [77] found that Sprague Dawley rats with ad libitum access to commercial Harlan ground 2020X supplemented with 0.1% *w*/*w* triclocarban during gestation and lactation exhibited significant changes in the community structure of the fecal microbiota, as well as finding decreased phylogenetic diversity in exposed dams and neonatal rats. Marked differences in β-diversity were found in exposed animals compared with controls in dams at 18 days of gestation and 16 day old neonates. This dose was chosen as it has been demonstrated that the serum TCC concentration of pregnant rats after oral exposure to 0.2% *w*/*w* TCC was similar to the concentrations reported in the human serum [146].

Ma et al. (2020) [78] studied the long-term effects in adult and old rats of perinatal exposure to TCs and found that 50 mg/kg/day (the lowest toxic oral dose in rats) resulted in disturbances of the metabolism and gut microbiota that were long-lasting and persisted even after the exposure had been terminated. They also accumulated over time, inducing metabolic disorders in old rat offspring. Exposure to TCs induced an increased growth of *Bacteroidetes*, which has been related to lipid accumulation [42]. Additionally, a reduction in *Akkermansia muciniphila,* a species linked to improved metabolism in diabetic and obese mice, was observed [147]. 

Interestingly, probiotics have been used to modulate the microbiota and palliate intestinal metabolic disorders due to triclosan exposure in animal models [80]. In this respect, *Lactobacillus plantarum* ST-III has been found to increase the diversity of the gut microbiota in zebrafish, thereby reducing the toxicity of chronic exposure to triclosan. Additionally, a probiotic-rich diet reduced the risk of lipid-metabolism disorders such as increased triglyceride and total cholesterol levels. Histopathological studies demonstrated severe structural damage to the intestines, spleen, and kidney after triclosan exposure; however, this damage can be reduced by the presence of *Lactobacillus*.

Bever et al. (2018) [85] compared the fecal microbiome of infants fed with breast milk that had measurable levels of TCS versus infants fed with breast milk that had no detectable concentrations of TCS and found that early life exposure to exogenous contaminants induces changes to microbiome diversity. Because of the impact of a healthy infant gut microbiome on phenotypes later in life, understanding how EDCs influence the infant gut microbiome is critical to identifying and correcting problematic changes in infant gut health. 

However, Ribado et al. (2017) [86] did not find that exposure to household TC-containing products induces changes to or a loss of microbial diversity, but they found increased *Proteobacteria* spp. in infants and mothers exposed to higher TC levels. Interestingly, increased *Proteobacteria* has been proposed as a potential diagnostic marker for dysbiosis and an increased risk of diabetes and colitis [148].

#### 3.2.8. Phthalates

Phthalates are EDCs used as plasticizers in food processing and packaging, adhesives, personal care products, and cosmetics. A major source of phthalate exposure is the diet, primarily due to contamination during processing and packaging [149]. Phthalates have been considered obesogens, therefore contributing to overweightness and obesity. It has been shown that exposure to phthalates alters glucose and lipid metabolism, which increases the risk of developing insulin resistance [150,151].

A mouse model of prenatal di(2-etilhexil) ftalato (DEHP) exposure (0.2, 2, and 20 mg/kg/day) was used to study the long-term metabolic disturbances in offspring. In an ICR mouse model of prenatal DEHP exposure (0.2, 2, and 20 mg/kg/day), Fan et al. (2020) [67] showed that exposure to low-dose phthalate (0.2 mg/kg/day) in mice induced changes in glucose metabolism, energy expenditure, adipogenesis, and gut dysbiosis in a sex-dependent manner. The level of DEHP exposure was selected based on the EPA reference dose. Their findings strengthen the hypothesis that connections between the host and gut microbiota alter energy metabolism.

As mentioned above, Hu et al. (2016) [66] reported that postnatal, low-dose exposure to diethyl phthalate (DEP) in Sprague-Dawley rats from birth through adulthood induced changes in the composition of the gut microbiota, but these changes were seen only in adolescent rats. The changes include an increased relative abundance of *Bacteroidetes* (*Prevotella*) *Elusimicrobia* and decreased *Firmicutes* (*Bacilli*) in exposed rats versus controls. Surprisingly, these DEP-induced changes decreased in adulthood despite continuous exposure, which suggests that the effects of exposure to environmental chemicals are more severe in adolescents. They also observed a small but consistent reduction of body weight in exposed adolescent rats, which is consistent with their findings of a reduced *Firmicutes*/*Bacteroidetes* ratio.

Lei et al. (2019) [37] conducted in vivo and in vitro experiments in female C57BL/6J mice to determine the effects of low or high dose DEHP (1 or 10 mg/kg bw/day) exposure on the gut microbiota composition and metabolite profile. The authors observed an increased abundance of *Lachnoclostridium* and decreased *Clostridium sensu stricto* after DEHP exposure. The addition of DEHP to the cultured cecal microbiota enhanced the abundance of *Lachnoclostridium,* which is able to produce p-hydroxyphenylacetic acid, the precursor of p-cresol, a bacterial metabolite linked to neurodevelopmental disorders.

Regarding human epidemiological studies, the information is scarce. Yang et al. (2019) [83] demonstrated in a recent epidemiological study showing that DEHP exposure in newborns resulted in changes to the microbiota composition, with a decrease in *Rothia* sp. and *Bifidobacterium longum*. The presence of *Rothia* in human milk has been associated with a minor incidence of asthma [152], and *B. longum*, considered a probiotic, seems to have positive effects on infants, particularly in reducing the risk of obesity and celiac disease. 

## 4. Conclusions

The incidence of metabolic diseases such as obesity and T2D are increasing worldwide. Exposure to EDCs related to food intake induces a series of changes including microbial dysbiosis and the induction of xenobiotic pathways and associated genes, enzymes, and metabolites involved in EDC metabolism. The products and by-products released following the microbial metabolism of EDCs can be taken up by the host and could have a major impact on host metabolism and the development of metabolic diseases. However, data regarding the effects of EDCs on the human gut microbiota are limited. The increasing EDC exposure via dietary intake requires the identification of the compounds and of the specific responses of the different species of the gut microbiome. In addition, the characterization of the common mechanisms of action of the different EDCs—such as the binding of the same hormone receptors, their possible cumulative and combined effects, and the indicative bacteria underlying the toxicity of EDCs on the gut microbiota—is also essential. In addition, the effect of exposure to low EDC levels on microbiota disruption should also be considered. The impact of other parameters such as host age and sex on the gut microbiota, described in this review, make necessary further research with broader dose ranges and analyses with more time points. This will help to determine the origin of sex-dependent effects using additional exposure windows. Lastly, the remediation of EDC-induced changes in the gut microbioma might represent an alternative for the treatment and prevention of metabolic diseases.

## Figures and Tables

**Table 1:** Effects of endocrine disruptors on the gut microbiota in in vitro assays.

References	Compound	Dose Exposure	Justification of Exposure Dose	Species Strain Mode	Methods	Outcomes	Conclusions
Van de Wiele et al. (2010) [35]	Metal (Arsenic)	10 μg methylarsenical/g biomass/hr and 28 μg as-contaminated soils/g biomass/hr	Concentrations detected in arsenic contaminated soils in urban areas of the EEUU	Strains isolated from human feces	HPLC; plasma mass spectrometry	High degree of methylation of Methylarsenical and As-contaminated soils in colon digestion.	Human microbiota has ability to actively metabolize As into methylated arsenicals and thioarsenicals.
Wang et al. (2018) [28]	BPA	25 μg/L, 250 μg/L, and 2500 μg/L	High human relevant exposure dose; EPA reference dose; 1% lowest observed adverse effect level	Humans	In vitro SHIME, 16S rRNA gene sequencing, and PCR	BPA exposure decreased the diversity of gut microbioma (ascending colon and the transverse colon). Exposure to BPA of 25 μg/L decreased diversity of gut microbioma, but high-level exposures (250 and 2500 μg/L) increased diversity (descending colon).	Exposure to BPA significantly altered the microbiota and increased the proportion of shared microbes.
Hoffman et al. (2019) [36]	PCB126	20 or 200 μM	Concentrations physiologically relevant, especially in heavily exposed populations	C56BL6/J mice	16S rRNA gene sequencing, PCR, and HPLC	Significant reduction in bacterial growth after exposure to high concentrations of PCB 126 compared to control. Not significant reduction in bacterial growth at PCB concentrations below 20 µM.	Exposure to PCB126 can contribute to alterations in host metabolism through mechanisms dependent on the intestinal microbiota, specifically through bacterial fermentation or membrane disruption.
Lei et al. (2019) [37]	Di (2-ethylhexyl) phthalate	10 or 100 µM	The concentration mimics human exposure during adolescence by continually exposing mice to phthalate from ages 6 to 8 weeks	C57BL/6J mice	16S rRNA gene sequencing and a triple-quadrupole time-of-flight instrument coupled to a binary pump HPLC system	Exposure of in vitro cecal microbiota to di (2-ethylhexyl)-phthalate increased the abundance of *Alistipes, Paenibacillus*, and *Lachnoclostridium*. Non-directed metabolomics showed that di (2-ethylhexyl)-phthalate greatly altered the metabolite profile in the culture.	Di (2-ethylhexyl)-phthalate can directly affect the production of bacterial metabolites related to neurodevelopmental disorders.
Joly et al. (2013) [38]	Chlorpyrifos	1 mg/kg/day	NOAEL	Wistar rats	SHIME	Exposure to chlorpyrifos increased *Bacteroides* spp. and *Enterococcus* spp. and reduced *Bifidobacterium* spp. and *Lactobacillus* spp.	Chronic, low-dose exposure to chlorpyrifos causes gut dysbiosis.
Shehata et al. (2013) [39]	Glyphosate	5.0, 2.40, 1.20, 0.60, 0.30, 0.15, and 0.075 mg/mL	To determine the minimal inhibitory concentration	Chickens	MALDI–TOF MS analysis, multiplex PCR	In vitro exposure to glyphosate showed resistance to glyphosate in highly pathogenic bacteria, but most beneficial bacteria showed susceptibility to glyphosate.	Glyphosate exposure showed differences in sensitivity between pathogenic and beneficial microbiota. Ingestion of glyphosate-contaminated food reduced the beneficial microbiota.
Ackermann et al. (2015) [40]	Glyphosate	0, 1, 10, and 100 μg/mL	Concentrations lower than NOAEL	Cows	DAISYII-incubators, FISH with 16S rRNA/23S rRNA-targeted	Exposure to 1 and 10 μg/mL glyphosate reduced abundances of all species except for *Isotricha* spp. and *Diplodinium* spp. Exposure to 100 μg/mL glyphosate reduced abundance of *Diplodinium* spp.	Glyphosate inhibits growth of beneficial bacteria, but increases the population of pathogenic bacteria
Riede et al. (2016) [41]	Glyphosate	0.42 or 2.92 mg/L	The low dose reflects the estimated maximum dietary glyphosate intake of dairy cattle, according to model assumptions. The high dose is higher than residues found in the beef cattle diet.	Cows	RUSITEC experiments, LC-MS/MS method, 16S rRNA gene sequencing, and PCR	Effects of glyphosate at concentrations of 0.42 or 2.92 mg/L. After the incubation period only observed subtle changes in the composition of ruminal bacteria.	No major changes were observed due to Glyphosate exposure to ruminal metabolism or the composition of bacterial communities.

HPLC: high performance liquid chromatography; PCR: polymerase chain reaction; SHIME: simulator of the human intestinal microbial ecosystem; FISH: fluorescence in situ hybridation; MALDI-TOF MS: matrix-assisted laser desorption/ionization time-of-flight mass spectrometry; RUSITEC: rumen simulation technique; NOAEL: no observed adverse effects level.

**Table 2:** Effects of endocrine disruptors on the animal microbiota.

References	Compound	Dose Exposure	Justification of Exposure Dose	Species Strain Mode	Methods	Outcomes	Conclusions
Ba et al. (2017) [42]	Metals (Cadmium)	100 nM	Tolerable weekly intake.	C57BL/6J mice	16S rDNA sequencing. Fecal microbiota transplant.	Early exposure to low dose of cadmium results in adiposities in adult male mice as well as in reduced diversity and altered composition of gut microbiota.	Early exposure to cadmium resulted in increased fat deposits in male but not in female mice. Low cadmium concentrations increased the expression of genes related to lipid metabolism.
Wu et al. (2016) [43]	Metals (Lead)	32 ppm	Relevant concentration of Pb acetate in drinking water.	Non-agouti (a/a) AVy mice offspring	16S rRNA gene sequencing.	Perinatal Pb exposure was significantly associated with increased bodyweight in adult males (*p* < *0*.05) but not in females (*p* = *0*.24). Perinatal Pb exposure altered gut microbiota composition in adult offspring, even after stopping exposure at 3 weeks (sex-independent). *Pseudomonas*, *Enterobacter*, and *Desulfovibrio* increased in adult mice perinatally exposed to Pb (*p* < *0*.05).	Perinatal Pb exposure was associated with bodyweight (sex-dependent response) and with microbiota composition changes (sex-independent).
Xia et al. (2018) [44]	Metals (Lead)	10 and 30 μg/L	Exposure dose is below the maximum allowable concentration of lead in water for zebrafish = 0.07 mg/L.	Zebrafish	16S rRNA gene sequencing and GC/MS metabolomics analysis.	Exposure to 30 μg/L Pb resulted in decrased *α-Proteobacteria* and increased *Firmicutes*. GC/MS metabolomics analysis showed that 41 metabolites were altered in the exposed group. Changes were related to glycolysis and lipid, amino acid metabolism, and nucleotide metabolism.	Pb exposure at 10 and 30 μg/L during 7 days was associated with changes in microbiota and in glucose, lipid, amino acid, and nucleotide metabolism.
Lu et al. (2014) [45]	Metals (Arsenic)	10 ppm as sodium arsenite	The exposure dose is above the maximum allowable concentration of arsenic in food.	C57BL/6 mice	16S rRNA gene sequencing and MS–based metabolomics profiling.	The most abundant gut bacteria were *Firmicutes* (52.79%) and *Bacteroidetes* (41.57%), followed by *Tenericutes* (3%), *Actinobacteria* (0.18%), *Cyanobacteria* (0.023%), and *Proteobacteria* (0.0042%)	Altered gut bacteria were strongly linked to changes in the microbiota metabolites. These changes increase the risk of tissue dysfunctions that might lead to obesity, insulin resistance, and cardiovascular disease.
Catron et al. (2019) [29]	BPA, BPAF, BPB, BPF, and BPS	BPA (0, 0.2, 0.6, 1.7, 2.9, 5.7, 11.5, 23.0, or 45.0 μM), BPAF (0, 0.2, 0.6, 1.8, 5.2, 15.3, or 45.0 μM), BPB (0, 0.6, 1.7, 5.1, 15.0, or 44.0 μM), BPF (0, 0.2, 0.6, 1.8, 5.2, 15.3, or 45.0 μM), or BPS (0, 0.2, 0.6, 1.8, 5.2, 15.3, or 45.0 μM)	Dose based on zebrafish toxicity data available through the iCSS ToxCast dashboard and previous zebrafish studies.	Zebrafish	16S rRNA gene sequencing.	Exposure to all the tested concentrations of BPS resulted in non-detectable levels of the *Neisseriaceae* family. Increasing BPS concentrations were associated with increased abundances of *Cryomorphaceae*. Increasing BPA or BPF concentrations were associated with increased abundances of *Chromatiaceae* and decreased abundances of *Neisseriaceae*.	BPS, BPA, or BPF exposure led to structural microbiota disruption during early development at concentrations not related to evident developmental toxicity. Results show that microbiota is very useful for characterization of health effects associated with exposure to environmental chemicals.
Chen, et al. (2018) [30]	BPA	0, 2, and 20 μg/L	Environmental concentrations.	Zefrafish (*Danio rerio*)	16S rRNA gene sequencing.	Nano-TiO_2_ and BPA co-exposure led to altered composition of guy microbiota with increased *Proteobacteria* and *Actinobacteria* in males and females. *Hyphomicrobium* was the most abundant genus in males and females.	Co-exposure to nano-TiO 2 and BPA modifies gut microbiome dynamics, having toxicological effects on host health.
DeLuca et al. (2018) [46]	BPA	50 µg/kg bw	Lowest observed adverse effect level.	C57BL/6 mice	IMAC at Texas A&M University and triple quadrupole mass spectrometer coupled to LC.	Exposure to BPA increased mortality, disease activity, and scores of colonic inflammation colon after exposure to sodium dextran sulfate.	BPA exposure decreased microbiota metabolites derived from aromatic amino acids and associated with colon inflammation and inflammatory bowel disease.
Javurek et al. (2016) [31]	BPA	50 mg/kg bw	Environmental exposure.	California mice	16S rRNA gene sequencing.	BPA exposure increased growth of pathogenic bacteria (*Bacteroides*, *Mollicutes*, and *Prevotellaceae*, among others) associated with inflammatory bowel disease, metabolic disorders, and colorectal cancer. However, increased *Bifidobacterium* was also found after BPA exposure.	Gut microbiota disruption secondary to BPA exposure is associated with systemic effects, such as inflammatory bowel disease, metabolic disorders, and colorectal cancer.
Koestel et al. (2017) [47]	BPA	52.2 ± 19.3 ng/food can or 36.2 ± 18.6 ng/food can	BPA levels identified in cans of diet. Serum dog concentrations 2.2 ng/mL, similar to that which has been reported in humans.	Dogs	16S rRNA gene sequencing.	Exposure to high concentrations of BPA was associated with increased bicarbonate levels in plasma and with changes in fecal microbiota (increased *Clostrididiaceae*, *Bacteroides* spp., *Clostridiales*, *Ruminococcus* spp., *Lachnospiraceae*, *Roseburia* spp., *Clostridium hiranonis*, and *Megamonas* spp.).	Exposure to high concentrations of BPA was associated with decreased *Bacteroides* spp., which is related to reduction in bacterial bisphenol degradation. This increases active BPA available for absorption in the gut.
Lai et al. (2016) [48]	BPA	Unknown concentration (BPA content in contaminated diet)	n.a.	CD-1 mice	LC-MS/MS, 16S rRNA gene sequencing, and amplicon PCR.	BPA and high-fat diet promoted growth of *Proteobacteria* (indicator of dysbiosis). Increased *Helicobacteraceae* proliferation and reduced *Firmicutes* and *Clostridia* were found in exposed mice.	Exposure to BPA in the diet led to structural changes in gut microbiota similar to those induced by high-fat diet and high-sucrose diet.
Liu et al. (2016) [32]	BPA	2000 μg/L	Dose used to simulate environmental exposure for a short period of exposure.	Zebrafish	16S rRNA gene sequencing and amplicon PCR reaction.	BPA exposure significantly modified gut microbiota composition with increased CKC4 phylum in male and female zebrafish.	BPA exposure altered gut microbiota composition. Gut dysbiosis may be related to changes in lipid metabolism of the host (increased triglycerides in the muscle).
Malaise et al. (2017) [5]	BPA	50 µg/kg bw	Exposure dose is 100 times below the current NOAEL in mice. NOAEL= 5 mg/kg BW/day	Mice	16S rRNA gene sequencing and Real time PCR.	Perinatal oral exposure to 50 µg/kg BPA led to gut and systemic immune changes in post-natal day 45. These changes were linked to altered glucose sensitivity and secretion of IgA in feces and decreased fecal *Bifidobacteria* compared to mice in the control group. These effects appear before the infiltration with proinflammatory M1 macrophages in gonadal white adipose tissue that appears with aging, along with decreased insulin sensitivity (T1D) and weight gain.	The results explain the sequence of changes related to perinatal exposure to BPA which could also explain the development of metabolic diseases in adulthood (decreased insulin sensitivity and increased weight gain).
Reddivari et al. (2017) [49]	BPA	200 μg/kg bw	To ensure gestational and lactational exposure of pups, approximately 1/25 of the NOAEL dose.	Rabbits	16S rRNA gene sequencing.	BPA exposure induced significant decrease in *Oscillospira* and *Ruminococcaceae* and therefore in short-chain fatty acid production. BPA exposure also reduced fecal levels of short-chain fatty acid, and increased systemic lipopolysaccharide and gut permeability.	Perinatal exposure to BPA modified gut microbiota composition and decreased beneficial bacterial metabolites such as short-chain fatty acids. BPA exposure also increased chronic inflammation in colon and liver, and systemic lipopolysaccharides.
Xu et al. (2019) [33]	BPA	30 or 300 µg/kg bw	Based on human exposure (30 µg/kg) and median human blood (300 µg/kg) levels	Mice	16S rRNA gene sequencing and amplicon PCR reaction.	BPA exposure-induced changes in gut microbiome composition are a potential mechanism of immunomodulation and T1D development. BPA at 30 or 300 µg/kg increased *Bacteroidetes*, and 300 µg/kg increased *Cyanobacteria* and TM7. The 30 µg/kg dose decreased *Proteobacteria*, and 300 µg/kg decreased *Firmicutes* and *Tenericutes*. Females showed an increase in pro-inflammatory factors, while males showed an increase in anti-inflammatory immune factors.	Altered gut microbiota and inflammation risk factors for T1D development associated with BPA exposure were sex-dependent.
Xu et al. (2019) [6]	BPA	30 or 300 µg/kg bw	To accelerate Diabetes type I development (30 µg/kg bw) and alter the immunity (300 µg/kg bw).	Mice	16S rRNA gene sequencing and amplicon PCR reaction	Adult females showed a higher risk of T1D and increased immune responses. However, female offspring showed lower risk of T1D and a shift towards anti-inflammation. In contrast, BPA exposure had little impact on DT1 and immunity in male offspring.	BPA effects on the development of T1D were related to host age and gender. Changes in gut microbiota and inflammation are responsible for T1D in juvenile exposure. Decreased inflammation is responsible for attenuated T1D in males and female offspring exposed during the perinatal period.
Chen, et al. (2018) [50]	PCB126 and PCB153	1.0 μg/L	Concentration below the limit established by EPA.	Zefrafish	16S rRNA gene sequencing.	PCB126 exposure led to altered microbiota and deterioration of the intestinal and hepatic functions. PCB126 was associated with oxidative stress and with a sexual dysmorphic effect. Exposure to PCB126 was significantly associated with oxidative stress, and exposure to PCB153 was associated with lower body weight, higher hepatosomatic index in female zebrafish, but lower index value in exposed males PCB153.	Exposure to PCB126 showed a significant correlation between dysbiosis and fish health.
Cheng et al. (2018) [51]	PCBs	6 or 30 mg/kg	To study dose-dependent effect of xenobiotics on the expression of hepatic drug metabolizing enzymes.	C57BL/6 mice	16S rRNA sequencing, quantitative PCR, and UPLC-MS/MS	Exposure to 6 mg/kg PCBs greatly increased the bacteria related to the metabolism of bile acids. This was associated with increased bile acids in serum and small intestine content in a microbiota-dependent fashion. However, at 30 mg/kg PCBs, bile acid levels remained stable and were linked with increased hepatic flow transporters and ileal Fgf15.	Changes in microbiota promoted increase in taurine mediated by PCBs in the muricholic acids α and β conjugated with taurine in liver, large and small intestine.
Chi et al. (2019) [52]	PCB126	50 μg/kg bw	Dose environmentally relevant to concentrations historically reported in lake trout from the Great Lakes.	C57BL/6 mice	16S rRNA gene sequencing and qRT-PCR	Exposure to PCB126 induced gut dysbiosis (increased *Firmicutes* and *Bacteroidetes* and decreased *Erysipelotrichia*) as well as dyslipidemia and nonalcoholic fatty liver disease.	Exposure to low doses of PCB-126 in mice caused intestinal microbiota dysbiosis and multiple disorders in serum and liver.
Choi et al. (2013) [53]	PCB153, PCB138, and PCB180	150 µmol/kg	PCB content in contaminated food.	C57BL/6 mice	16S rRNA gene sequencing and PCR.	Exposure to PCBs in sedentary mice resulted in decreased abundance of *Proteobacteria*. Exercised mice showed a gut microbiome structure significantly different from sedentary mice. Exercise lessened PCB-induced changes in gut microbiome.	Exposure to PCBs promotes changes in gut microbiome, which can determine systemic toxicity. Physical exercise lessens changes in gut microbiome.
Kohl et al. (2015) [54]	PCB126	7.3 ng/g	The exposure concentration is below the maximum allowable concentration of PCBs in food.	Northern leopard frogs (*L. pipiens*)	16S rRNA gene sequencing	Tadpoles exposed to PCB126 maintained increased *Fusobacteria* (t = 2.95; df = 14; *p* = 0.01). *Fusobacteria* was a very small portion of the tadpole (average of control and treated with PCB: 0.008%) and control frog (0.3 ± 0.1%) gut communities. Frogs exposed to PCB126 during larval stage had a relative Fusobacterial abundance of 3.5 ± 1.4%.	Exposure to PCB126 results in changes in gut microbiota communities, which might affect health and fitness of the host.
Petriello et al. (2018) [55]	PCB126	1 μmol/kg	This dose produces plasma PCB 126 levels that mimic human exposures of dioxin-like pollutants.	*Ldlr* −/− mice	16S rRNA gene sequencing and regression modeling.	PCB126 reduced α diversity (*p* = 0.001) in the colon and increased the *Firmicutes* to *Bacteroidetes* ratio (*p* = 0.044). Quantifiable amounts of PCB126 in the colon, upregulation of Cyp1a1 gene expression, and increased indicators of gut inflammation were found in exposed mice.	PCB126 exposure altered gut microbiota and metabolism and resulted in gut and systemic inflammation.
Rude et al. (2019) [56]	PCBs	0.1, 1, or 6 mg/kg/day	FDA mandates tolerances of 0.2–3.0 ppm (200–3000 ng/g) for all foods.	Mice	qPCR and 16S rRNA gene sequencing.	PCB exposure resulted in epithelial permeability defects in the ileum and colon of juvenile mutated mice. PCB exposure also promoted intestinal inflammation dysbiosis in gut microbiota in juvenile mutated mice exposed to 1 mg/kg/d PCBs versus controls.	The results showed the interactions between PCBs and genetic susceptibility factors to impact individual risk.
Horiuchi et al. (2017) [57]	Phytoestrogen (S-equol)	20 mg/kg, 2 times/d	Doses based on previous studies of the possible benefits of S-equol on diabetes.	Mice	Immunochemistry. Insulin secretion assay. qRT-PCR.	Administration of S-equol resulted in reduction of the induction of blood glucose concentrations (*p* < 0.01 at 15 min, *p* < 0.01 at 30 min, *p* < 0.05 at 60 min, and *p* < 0.01 at 120 min)	Gut microbiota-produced S-equol induced β-cell growth in vivo and insulin secretion ex vivo. Administration of S-equol decreased *Streptozotocin*-induced hyperglycemia by promoting β-cell function.
Huang et al. (2018) [58]	Phytoestrogens (Genistein)	20 mg/kg bw	Dose physiologically relevant to obtain an accurate interspecies extrapolation.	Mice	16S rRNA gene sequencing and qRT-PCR.	Perinatal genistein exposure caused increased incidence of DT1 in female offspring. Fecal microbiota from female offspring at postnatal day 90 showed increased *Enterobacteriales* (suggesting a proinflammatory response). In contrast, perinatal genistein administration caused a shift in microbiota towards anti-inflammation in males at postnatal day 90.	Perinatal administration of genistein resulted in strongly sex-dependent changes in microbiota. T1D exacerbation in non-diabetic females was related to immunomodulatory mechanisms associated with an altered gut microbiota.
López et al. (2018) [59]	Phytoestrogens (Genistein)	3 mg/kg bw/day	Equivalent dose of 1.5 g of genistein per day for a 65 kg adult person, approximately.	C57BL/6 mice	16S rRNA gene sequencing and qRT-PCR.	Mice fed with a high-fat diet with genistein exhibited changes in the gut microbiota linked to lower circulating levels of lipopolysaccharides, improved glucose metabolism, and reduced expression of pro-inflammatory cytokines in the liver compared to mice in the high-fat diet alone group.	Genistein exposure through diet can modulate gut microbiota, decreasing metabolic endotoxemia and neuroinflammatory response despite consumption of a high-fat diet.
Marshall et al. (2019) [60]	Phytoestrogens (Genistein)	250 mg/kg	The dose is above the maximum allowable concentration of genistein in food. EFSA LOAEL of 35 mg/kg bw/day for males and 44 mg/kg bw/day for females	California mice	GC/MS, 16S rRNA sequencing Social behavior testing using the three-chamber test	When male offspring from genistein-supplemented dams were compared with genistein-free offspring, audible calls above 20 kHz correlated with daidzein, α-tocopherol, *Flexispira* spp., and *Odoribacter* spp. Results suggest that early genistein exposure can induce a disruption in the offspring’s normal socio-communicative behaviors.	Perinatal exposure to genistein may detrimentally affect the offspring “microbiome-gut-brain axis”.
Piccolo et al. (2017) [61]	Phytoestrogens	Phytoestrogens naturally present in diet (pigs fed with soy-based infant formula)	Food exposure levels.	White Dutch Landrace pigs	16S rRNA gene sequencing and LC-MS.	Sow-fed piglets exhibited higher α-diversity in the duodenum than formula-fed piglets (*p* < 0.05). No differences were found in the ileum. *Firmicutes* was the most abundant phylum in the duodenum in all tested diets, followed by *Proteobacteria* in the sow-and milk-fed piglets and *Cyanobacteria* in soy-fed piglets.	Neonatal diet can impact small intestine microbiome in pigs, resulting in disturbances in the metabolism and development of intestinal tissue in the postnatal period.
Williams et al. (2019) [62]	Phytoestrogens	Phytoestrogens naturally present in diet	Food exposure levels.	Southern white rhinoceros (*Ceratotherium simum simum*)	16S rRNA amplicon sequencing and MS.	Composition and structure of fecal microbiota significantly differs by rhino species as well as at the phylum, family, and OUT levels.	Results suggest differences in receptor sensitivity to phytoestrogens related to the species and metabolism of dietary phytoestrogens by gut microbiota might have an impact on fertility of captive female rhinos.
Yeruva et al., (2016) [63]	Phytoestrogens	Phytoestrogens naturally present in diet (pigs were fed soy or milk formula).	Food exposure levels.	White Dutch, Landrace Duroc pigs	16S rRNA amplicon sequencing, qRT-PCR, ELISA and UHPLC-HRAM.	In soy-fed piglets, increased *Lactobacillaceae* spp. and *Clostria* spp. but decreased *Enterobacteriaceae* spp. were observed.	Neonatal diet promotes disturbances in microbiome of the small intestine in pigs, particularly in the duodenum.
Zhou et al. (2018) [64]	Phytoestrogens (Genistein)	0.25 and 0.6 g/kg	To analyze the effects of low and high exposure doses.	C57BL/6 mice	Oral glucose tolerance tests, ELISA kit, and 16S rRNA amplicon sequencing.	Perinatal maternal consumption of a high-fat diet with genistein resulted in increased birth weight, improved glucose tolerance and decreased fasting insulin, as well as lower levels of triacylglycerol and total cholesterol in serum in the offspring.	The intake of genistein during pregnancy improves the metabolism of the offspring, preventing the transgenerational effects of maternal high-fat diet on diabetes.
Obadia et al. (2018) [65]	Methylparaben (MPB)	0.0, 0.1, 0.2, and 0.3%	High levels of MPB (∼ 0.5%) can affect the microbiota diversity; the exposure dose was selected to test lower levels.	*Drosophila melanogaster*	-	Concentrations > 0.1% MPB disrupt the growth of some species of yeast and *Acetobacter*, but even at 0.3% *Lactobacilli* growth was less affected.	Exposure to MPB probably alters the composition and amount of gut bacteria and yeasts in laboratory fly.
Hu et al. (2016) [66]	Diethyl phthalate (DEP), methylparaben (MPB), triclosan (TCS) and their mixture (MIX)	0.050 mg TCS/kg bw, 0.1050 mg MPB/kg bw and 0.1735 mg DEP/kg bw	NOAEL.	Sprague-Dawley rats	16S rRNA gene sequencing and PCR.	Exposure to these chemicals produced an increase in *Bacteroidetes* (*Prevotella*) and decreased *Firmicutes* (*Bacilli*) in all the exposed rats. Increased *Elusimicrobia* was found in DEP and MPB exposed rats, Betaproteobacteria in MPB and MIX exposed rats, and *Deltaproteobacteria* in the TCS group. In adulthood, these differences decreased between cases and controls despite continued exposure, suggesting that contaminant exposure have a greater impact on gut microbiome of adolescent rats.	Exposure at doses similar to environmental human exposure can disturb the gut microbiota in adolescent rats. This disturbance mainly affects the health of the youngest.
Fan et al. (2020) [67]	Di (2-ethylhexyl)-phthalate	0.2, 2, and 20 mg/kg/day	Based on the EPA reference dose and previous studies.	Mice	LC-HRMS, 16S rRNA gene sequencing, and qPCR.	Prenatal exposure to low doses of di (2-ethylhexyl) phthalate (0.2 mg/kg/day) resulted in metabolic syndrome and gut dysbiosis. Thiamine liver metabolism was disrupted in the offspring, which caused disturbances in glucose metabolism.	Exposure to low doses of di (2-ethylhexyl) phthalate during the early stages of life might increase the risk of obesity and metabolic syndrome.
Lei et al. (2019) [37]	Di (2-ethylhexyl)-phthalate	1 or 10 mg/kg bw/day	The concentration mimics human exposure during adolescence by continually exposing mice to phthalate from ages 6 to 8 weeks.	C57BL/6J mice	16S rRNA gene sequencing and a triple-quadrupole time-of-flight instrument coupled to a binary pump HPLC system.	Oral probe di (2-ethylhexyl) phthalate exposure increased the abundance of *Lachnoclostridum*, while decreasing *Akkermansia*, *Odoribacter*, and *Clostridium* sensu stricto.	Di (2-ethylhexyl) phthalate exposure directly alters microbiota therefore modifying the production of bacterial metabolites related to neurodevelopmental disorders.
Gao et al. (2017) [68]	Diazinon	4 mg/L	According to previous studies, dose that did not elicit discernible AChE inhibition.	C57BL/6 mice	16S rRNA gene sequencing and mass spectrometry–based metabolomics.	Diazinon exposure significantly disturbed the intestinal microbiome, and the RNA sequencing revealed that diazinon exposure disrupts the functional metagenome. These changes were more pronounced male mice.	Diazinon exposure disturbed the structure of the gut microbiome, the functional metagenome and also had a sexual dysmorphic effect.
Jin et al. (2015) [69]	Carbendazim	100 or 500 mg/kg bw	The exposure concentration is above the maximum allowable concentration of carbendazim in food.	ICR mice (*Mus musculus*)	Real time PCR sequencing and 16s rRNA gene sequencing and HPLC.	Carbendazim exposure at 100 and 500 mg kg led to histopathological changes in the liver, disturbed lipid metabolism, and intestinal gut dysbiosis. During the first three days of exposure to carbendazim the most abundant constituents of microbiomes, *Firmicutes* and *Bacteroidetes*, tend to decrease. From the fifth day of treatment with carbendazim, *Bacteroidetes* maintained the decreasing tendency, but *Firmicutes* started to increase.	Exposure to carbendazim disturbs microbiota and can lead to inflammation, which results in altered lipid metabolism and triggers obesity in exposed mice.
Liang et al. (2019) [70]	Chlorpyrifos	5 mg/kg	Concentration higher than NOAEL.	C57Bl/6 and CD-1 mice	16S rRNA gene sequencing and qPCR.	Exposure to chlorpyrifos in mice induced changes in microbiota, increased body weight, and lowered insulin sensitivity. Chlorpyrifos also resulted in disruption of the intestinal barrier and more, which led to the entry of lipopolysaccharides in the body, which promote the release of pro-inflammatory factors.	Chlorpyrifos exposure might contribute to the worldwide epidemic of inflammatory diseases.
Liu et al. (2017) [71]	p, p’-dichlorodiphenyldichloroethylene (p, p’-DDE) and β-hexachlorocyclohexane (β-HCH)	1 mg p, p’-DDE/kg bw/day and 10 mg β-HCH/kg bw/day	These doses mimic the chronic exposure in human.	C57BL/6 mice	16S rRNA gene sequencing, real time PCR and UPLC-M.	Exposure to organochlorines disturbed the abundance and composition of gut microbiota (increased *Lactobacillus* capable of deconjugating bile salts). This affects the hydrophobicity and composition of bile acids, down-regulates the expression of genes involved in the reabsorption of bile acids in the distal ileum, and up-regulates the expression of genes involved in the hepatic synthesis of bile acids.	Chronic exposure to low doses of organochlorines increases the risk of dysfunction in bile acid metabolism.
Tu et al. (2019) [72]	2,4-Dichlorophenoxyacetic acid (2, 4-D)	< 15 mg/kg bw/day	Concentration lower than NOAEL.	C57BL/6 mice	16S rRNA gene sequencing and LC-MS.	Metagenomic results revealed a distinct intestinal microbiota with changes in various microbial metabolic pathways, including urea degradation, and amino acid and carbohydrate metabolism.	2,4-D exposure resulted in changes in the composition and activity of gut microbiota. The metabolic profile of host plasma samples showed changes in the metabolic profiles indicative of 2,4-D toxicity at low doses.
Yang et al. (2019) [73]	Organophosphorus: diethyl phosphate (DEP)	0.08 or 0.13 mg/kg	1/500 LD50.	Wistar rats	NanoDrop spectrophotometer and 16S rRNA gene sequencing.	Exposure to high dose of DEP promotes the growth of butyrate-producing bacterial genera Alloprevotella and Intestinimonas, which induced an increase in estradiol and a decrease in total triglycerides and low density lipoprotein cholesterol. Exposure to DEP also increased tyrosine-tyrosine peptide and ghrelin, attributed to the enrichment of *Clostridium* sensu stricto 1 and *Lactobacillus*, producers of short-chain fatty acids.	Chronic exposure to DEP affected the gut microbiota, serum hormones, and proinflammatory cytokines in rats, with stronger responses observed at high doses.
Wu et al. (2018) [74]	Propamocarb	0, 0.5, 5, 50 mg/kg bw/day	Dosages set according to the highest residue from the EU-MRLs and the NOAEL or long term toxicity.	Mice	16S rRNA gene sequencing.	Exposure to propamocarb disturbed the transcription of liver genes related to regulation of lipid metabolism. The microbiota in the cecal content and feces changed at the phylum or gender level.	Exposure to high dose propamocarb changes the metabolism of mice by altering the gut microbiota and microbial metabolites.
Gao et al. (2017) [75]	Triclosan (TCS)	2 ppm	The concentration used is 100 times lower than that which can promote liver carcinogenesis.	C57BL/6 mice	16S rRNA gene sequencing and shotgun metagenomics sequencing.	Exposure to TCS produced significant changes in mouse gut bacterial community assembly. Metagenomic sequencing showed an increase in gut bacterial genes related to triclosan resistance, stress response, and antibiotic resistance, and others.	Exposure to TCS alters the intestinal microbiome of mice by inducing changes at the compositional and functional levels.
Gaulke et al. (2016) [76]	TCS	100 μg/g	Dose to cause endocrine disruption in fish.	Zebrafish	16S rRNA gene sequencing.	Operational taxonomic units of the *Enterobacteriaceae* family are susceptible to TCS exposure, but operational taxonomic units of the *Pseudomonas genus* are more resistant to exposure.	Exposure to TCS promotes changes in the composition and ecological dynamics of gut microbial communities.
Kennedy et al. (2016) [77]	TCs (TCS and Triclocarban (TCC))	0.1% (*v*/*v*)	According to blood human level.	Timed-pregnant Sprague Dawley (SD) rats	16s rRNA gene sequencing.	TCC exposure reduced the diversity of fecal microbiota in exposed rats versus controls at 7 days after exposure. This continued throughout perinatal exposure.	α-diversity was reduced in exposed animals at all sampling time points after baseline. Differences in β-diversity were found between gestational day 18 and post-delivery day 16 in exposed versus control dams.
Ma et al. (2020) [78]	TCS	0, 10, or 50 mg/kg	Doses referenced previous toxicity studies in rats (Lowest toxic dose in rats is 50 mg/kg/day)	Rats	16S rRNA gene sequencing.	Exposure to TCS reduced diversity and altered the microbiota composition at doses of 50 mg/kg/day in adult rats and at two doses in old rats. These changes were long-lasting even after the exposure was terminated and accumulated over time, inducing metabolic disorders in old rat offspring.	Exposure to TCS early in life results in long-lasting changes in the metabolism and intestinal microbiota and they accumulate over time.
Narrowe et al. (2015) [79]	TCS	100 to 1000 ng/l	Environmentally relevant concentrations	Larval fathead minnows (Pimephales promelas)	High-throughput 16S rRNA sequencing	TCS resulted in an increase of all members of the order *Pseudomonadales*, in five *Acinetobacter* OTUs, and in 26 OTUs (*Flavobacterium*, *Chryseobacterium*, and *Shewanella*) at day 7.	Short-term, low-level environmental exposure to TCS is sufficient to disrupt gut microbiome in minnows.
Zang et al. (2019) [80]	TCS	0.002% (*v*/*v*)	Based on previous studies	Zebrafish (*Danio rerio*)	16S rRNA gene sequencing and qRT-PCR	TCS exposure led to severe structural and morphologic damage to the intestines, spleen, and kidney observed in histopathologic studies. *Lactobacillus* was able to mitigate this damage.	*Lactobacillus plantarum* ST-III increases gut microbial biodiversity in zebrafish and mitigates the damages associated with TCS exposure.

NOAEL: no observed adverse effects level; LOAEL: lowest observed adverse effect level; MRL: maximum residues levels.

**Table 3:** Effects of endocrine disruptors on the human microbiota.

References	Compound	Exposure Route	Species Strain Mode	Methods	Outcomes	Conclusions
Eggers et al. (2019) [81]	Metals (lead)	Environmental (food)	Adult humans	DNA sequencing of the 16S rRNA V4 region	Increased urine Pb levels were associated with the presence of *Proteobacteria*, increased α-diversity (*p* = 0.071), and wealth (*p* = 0.005). Changes in β-diversity were significantly associated (*p* = 0.003) with differences in Pb levels.	Pb exposure is associated with diversity and compositional changes of intestinal microbiota in adults.
Wu et al. (2016) [82]	Phytoestrogen	Oral (diet)	Adult humans	16S rRNA-tagged sequencing and plasma and urinary metabolomic platforms	Consumption of fermentable substrates was not associated with higher levels of short-chain fatty acids in fecal samples in vegans.	Despite the differences in plasma metabolome between vegans with high soy consumption and omnivores, the gut microbiota in the two groups was similar.
Yang et al. (2019) [83]	Phthalates (Di(2-ethylhexyl) phthalate)	Intravenous (plastic)	Newborns	Water ACQUITY UPLC and MS/MS; 16S rRNA sequencing	Biota differences were found between meconium samples and fecal samples collected later. Di(2-ethylhexyl) phthalate-exposed microbiota showed higher variability of bacteria taxa.	Short-term di(2-ethylhexyl) phthalate exposure led to temporary gut dysbiosis. This suggests that long-term exposure may result in permanent gut dysbiosis. Di(2-ethylhexyl) phthalate levels did not alter the dominant bacterial phyla composition, but the *Firmicutes-Bacteroidetes* ratio changed over time in both exposed and unexposed newborns.
Stanaway et al. (2017) [84]	azinphos-methyl	Oral and inhalation	Adult men	Isotope dilution GC-HR-MS, 16S rRNA gene DNA sequenced and Agencourt AMPure XP PCR purification system	Disturbances in *Streptococcus, Micrococcineae, Gemella, Haemophilus, Halomonas, Actinomycineae, and Granulicatell* were observed, and decreased oral bacterial genus *Streptococcus*.	Human exposure to agricultural pesticides is associated with the alteration of oral microbiota, but future research is needed to support these findings.
Bever et al. (2018) [85]	TCS	Oral (breast milk)	Infants and Mothers	16S rRNA sequencing and GC-MS	Diversity in fecal microbiome of TCS-exposed infants versus unexposed infants differed	Exogenous chemicals are correlated with disturbances in microbiome diversity in the intestinal community of infants during the early developing period.
Ribado et al. (2017) [86]	TCs (TCS and TCC)	Dermal (personal care products)	Infants and Mothers.	16s rRNA sequencing	TC exposure was not associated with a reduction of gut microbiota diversity in mothers and their infants at any of three time points after birth. Shannon’s diversity index was not decreased in infants randomized to TC-containing products.	After 10 months, chronic TC exposure from household products does not contribute to recovery of gut microbiomes in mothers or their infants. The most abundant species in the unexposed infants, *B. fragilis*, is associated with direct maturation of the immune system and the production of anti-inflammatory polysaccharides.
